# Intelligent weight prediction of cows based on semantic segmentation and back propagation neural network

**DOI:** 10.3389/frai.2024.1299169

**Published:** 2024-01-29

**Authors:** Beibei Xu, Yifan Mao, Wensheng Wang, Guipeng Chen

**Affiliations:** ^1^Agricultural Economics and Information Institute, Jiangxi Academy of Agriculture Sciences, Nanchang, China; ^2^Department of Population Medicine and Diagnostic Sciences, Cornell University, Ithaca, NY, United States; ^3^Department of Mathematics and Statistics, McMaster University, Hamilton, ON, Canada; ^4^Agricultural Information Institute, Chinese Academy of Agriculture Sciences, Beijing, China; ^5^Jiangxi Province Engineering Research Center of Intelligent Perception in Agriculture, Jiangxi Academy of Agriculture Sciences, Nanchang, China

**Keywords:** weight prediction, semantic segmentation, machine learning, computer vision, precision farming

## Abstract

Accurate prediction of cattle weight is essential for enhancing the efficiency and sustainability of livestock management practices. However, conventional methods often involve labor-intensive procedures and lack instant and non-invasive solutions. This study proposed an intelligent weight prediction approach for cows based on semantic segmentation and Back Propagation (BP) neural network. The proposed semantic segmentation method leveraged a hybrid model which combined ResNet-101-D with the Squeeze-and-Excitation (SE) attention mechanism to obtain precise morphological features from cow images. The body size parameters and physical measurements were then used for training the regression-based machine learning models to estimate the weight of individual cattle. The comparative analysis methods revealed that the BP neural network achieved the best results with an MAE of 13.11 pounds and an RMSE of 22.73 pounds. By eliminating the need for physical contact, this approach not only improves animal welfare but also mitigates potential risks. The work addresses the specific needs of welfare farming and aims to promote animal welfare and advance the field of precision agriculture.

## 1 Introduction

Modern society is concerned about food safety and quality, efficient and sustainable animal farming, healthy animals, and guaranteed animal welfare of livestock farms (Blokhuis et al., 2003; Berckmans, 2014). Livestock farming places high demands on both the farmers and their cow monitoring techniques, and these demands are likely exacerbated as farms increase in size (Robbins et al., 2016). Farms are always under constant pressure to be profitable which can be challenging in environments where labor costs are variable (MacDonald et al., 2007). In addition, livestock farms have also been challenged with managing disease, another factor that can impact farm efficiency. Specifically, due to the poor monitoring of livestock disease and impaired fertility in intensive dairy farming, the largest economic losses and cattle welfare can be seriously affected (Weary et al., 2009; Ashfaq et al., 2015; Daros et al., 2020).

Traditional farming methods usually monitor and treat the herd collectively according to the measured average ambient conditions. As farm sizes increase an additional challenge is the individual monitoring within the herd. Technical development of automatic monitoring of individual body conditions and health is of great interest, and it is important to find early indicators of diseases (Stern et al., 2015; Gu et al., 2017). Precision farming technologies that combine Artificial Intelligence (AI) with the Internet of Things (IoT) provide the potential to treat livestock individually, for the sake of better livestock welfare and production (Wathes et al., 2008; González et al., 2015; Norton and Berckmans, 2017). As such, automated and precise management of livestock, including the use of intelligent perception-based software, has been suggested by some scholars to be the next frontier in terms of monitoring individuals within a group (Qiao et al., 2021).

Previous research indicated that the individual cattle weight information is not only an important basis for live cattle trading, but also can be regarded as a key indicator for studying food conversion rate, individual daily weight gain, and setting feeding standards for cattle (Kohiruimaki et al., 2006; Berry et al., 2007; Poncheki et al., 2015). Typically, cows are routinely guided to the weighing systems through human intervention, which is bound to cause stress reactions and adverse effects on subsequent eating and growth (Charmley et al., 2006; Alawneh et al., 2011). In order to improve animal welfare, intelligent weighing methods are gradually populated with the help of measuring tools such as sensors and computer vision technology (Tasdemir et al., 2011; Nyalala et al., 2021; Sant'Ana et al., 2021). Generally, morphological traits information is extracted from images of cows to obtain relevant body size or area parameters. Subsequently, the weight of the cattle is accurately predicted based on the linear or nonlinear relationship between these parameters and weight (Cominotte et al., 2020; Dohmen et al., 2022; Li et al., 2022; Ruchay et al., 2022). Therefore, the use of computer vision for cattle weight prediction has advantages in automation, processing speed, and animal welfare.

In order to measure the morphological traits automatically, researchers selected and defined the back area, body size or fusing area, and height as the pre-identified features (Kuzuhara et al., 2015; Gjergji et al., 2020; Na et al., 2022). However, measurements based on the back area are susceptible to variations in cow postures. Moreover, the presented area can also be influenced by the distance between the camera equipment and the cows. Although the measurement of body size makes use of key areas or body parts like body width and height, heart girth, hip width, and height and thus achieves high accuracy, expensive equipment needs to be equipped at different aspects and angles accordingly, which is not applicable to housing farms (Qiao et al., 2019; Du et al., 2021; Zhang et al., 2021; Dang et al., 2022). Instead, the method integrating area and height takes advantage of three-dimensional size information of cows and becomes an accurate and reliable measurement, which is also consistent with the favorite indicators of experienced farmers for weight estimation artificially. For this purpose, besides the reference cards and image processing software (Ozkaya and Bozkurt, 2008; Weber et al., 2020a), different computer vision methods have been attempted to calculate the body areas and height including the Euclidean distances (Weber et al., 2020b), EfficientNet, ResNet, Recurrent Attention Model (Gjergji et al., 2020). Moreover, considering the strong correlation between the body parameters from the images and cattle weight, the regression-based machine learning methods, for instance, multiple linear regression (MLR) (Freund et al., 2006), support vector machine (SVM) (Boser et al., 1992), backpropagation (BP) neural network (Hakem et al., 2022) were used to predict the body weight.

In practical applications, traditional segmentation algorithms or machine vision algorithms face challenges in accurately extracting contours due to factors such as cubicle sheds, feces-contaminated ground, and variations in illumination. Overcoming these challenges is crucial to ensure the accuracy of body parameters. While previous studies have made significant contributions to cattle weight prediction in different ways, there is still a need for a comprehensive system that integrates low-cost hardware resources and enables accurate and automatic real-time weight estimation. The advancements in deep learning, particularly in semantic segmentation and instance segmentation technologies, present new opportunities for precise body image segmentation, thus facilitating the application of computer vision technology in livestock weight measurement (Borges Oliveira et al., 2021; Dohmen et al., 2021; Witte et al., 2021; Duan et al., 2023; Hou et al., 2023).

Semantic segmentation methods have demonstrated remarkable capabilities in image analysis tasks, especially in scenarios where precise delineation of object boundaries is essential. In the context of livestock weight measurement, semantic segmentation is particularly advantageous in providing a pixel-level understanding of the cow's physical structure. This study specifically employs semantic segmentation to extract fine-grained features, such as body shape and the precise positions of different body parts. This approach surpasses traditional segmentation methods by providing a pixel-level representation of the cow's physical structure. The resulting segmentation information enables the extraction of key body size parameters, including length, width, and height. By incorporating these detailed segmented parameters into weight prediction models, the aim is to enhance the accuracy of weight estimations by providing additional contextual information.

The motivation behind selecting semantic segmentation lies in its capability to offer high-resolution, detailed information about the cow's physical characteristics. This detailed information is instrumental in improving the precision of weight prediction models. By adopting state-of-the-art semantic segmentation models, the goal is to achieve a non-invasive, automated method for obtaining accurate body parameters. The subsequent integration of regression-based machine learning methods further refines weight predictions. The proposed approach aims to facilitate the automatic acquisition of objective sensory data from multi-view images, reducing the reliance on manual intervention while ensuring accurate and reliable weight predictions.

## 2 Materials and methods

### 2.1 Data collection and annotation

The experimental data of this study were collected from private farms in Jiangxi Province, China. The ages of cows ranged from 4 to 23 months, which were weighed using a scale to record their actual weight. The Sony FDR-AX40 camera was selected to capture the top-view and back-view images of 55 cows in the natural environment of barns. The top-view data was taken within the field of view of one cow body length where the camera was about 2.5 m from the ground and could be moved in the direction parallel to the cattle. The camera was fixed 1.5 m above the ground and 2.3 m away from the cows while collecting the back-view data, so that the horizontal field of view was 2–2.5 cattle width.

The resolution of top-view and back-view frames was 3,840 × 2,160 pixels. To reduce the equipment calculation, the frames were normalized in proportion and then resized to 704 × 1,216 pixels for top view and back view respectively. The dataset in this paper includes 550 images for top-view and 550 images for back-view, in which the training data and testing data for both top-view and back-view were randomly selected at a ratio of 8:2.

While the prediction of cattle body weight is intricately tied to various indicators such as body length, height, width, rump height, and rump width, individually calculating these indicators proves cumbersome (Dohmen et al., 2022; Zhao et al., 2023). Moreover, the automatic application of these calculations in breeding barns often leads to significant errors. Therefore, this paper takes the practical application into consideration and explores to integrate the body length, width, and hip width into the area in the top view and the body height as well as the hip height into the area in the back view of the cow body respectively.

**Figure 1:**
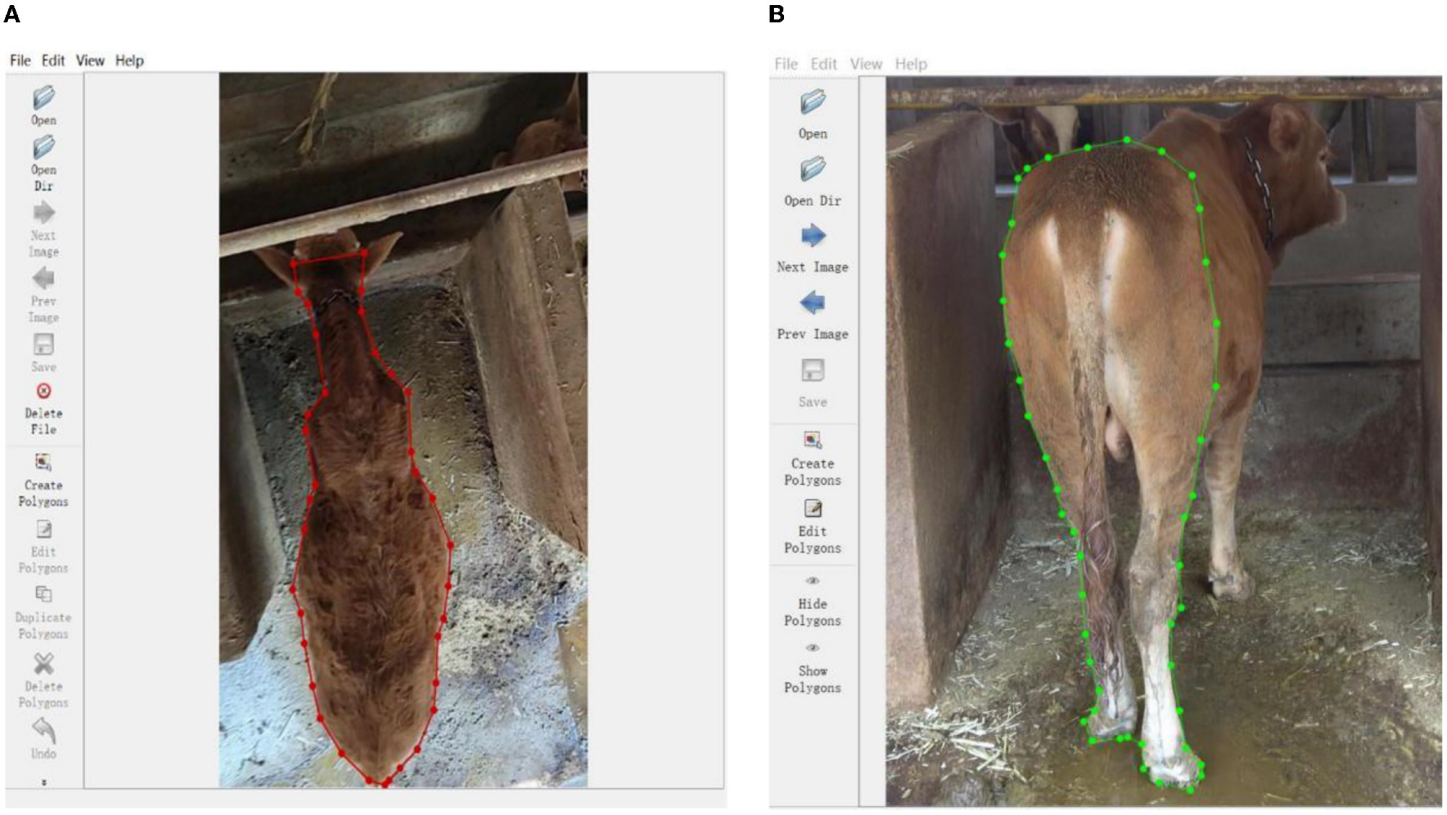
The image labeling of top view and back view. **(A)** Top-view. **(B)** Back-view.

Since the supervised semantic segmentation methods were used in this work, the graphical image annotation tool, Labelme, that supports annotation for semantic and semantic segmentation was used to label the contour of cows (Russell et al., 2008). When the json files generated by Labelme were converted to the mask files, the bites of mask files stored in 16 bits needed to be converted to 8 bites, which can be read by OpenCV in the model. Figure 1 shows the image labeling of top view and back view.

### 2.2 Semantic segmentation method for cow body parameters

**Figure 2:**
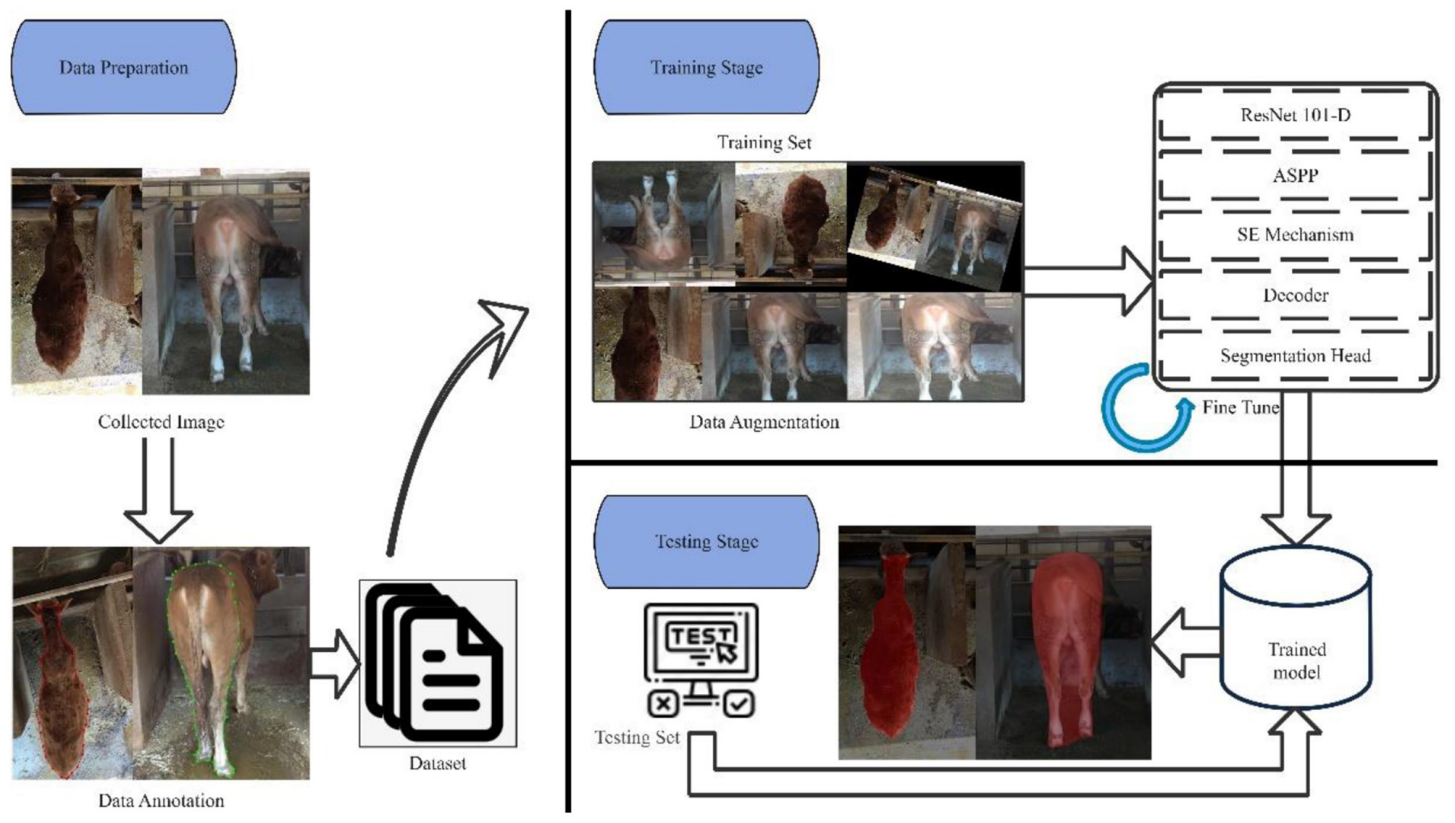
The proposed semantic segmentation model for body size parameters extraction.

The proposed method adopted in this paper built upon state-of-art semantic segmentation models to extract the cow body contours and obtain precise pixels of key parts. By leveraging the strengths of both semantic segmentation and object detection, the proposed semantic segmentation model employed the encoder-decoder architecture to integrate the high-level and low-level features of images and finally produced precise contour coordinates, object classes, and binary masks for predictive statistical parameters. The overview of the proposed pipeline is shown in Figure 2.

To improve the model's generalization and robustness, data augmentation techniques were implemented during the training stage on the annotated dataset. These techniques encompassed various transformations applied to the images, including rotation, scaling, and flipping, thereby simulating different viewing angles and orientations. By introducing such variations, data augmentation expanded the dataset and introduced diversity, enabling the model to learn effectively across various scenarios and enhance its performance on unseen data. The encoder and decoder components served as critical elements in the proposed model for processing the input image and generating a high-resolution semantic segmentation map. The encoder extracted high-level semantic features using a backbone, ResNet-101-D, and captured multi-scale contextual information through the employment of an Atrous Spatial Pyramid Pooling (ASPP) module (Chen et al., 2017). The decoder refined segmentation outcomes by fusing low-level spatial information from the early layers of the backbone with the high-level semantic information obtained from the ASPP module. Subsequently, the fused feature map undergone pixel-wise classification to yield a high-resolution semantic segmentation map.

#### 2.2.1 ResNet-101-D

The selection of a suitable model architecture is crucial for achieving high performance in computer vision tasks. In this regard, ResNet-101-D has been chosen due to its exceptional performance in various computer vision tasks and its ability to handle complex visual data (He et al., 2019). This architecture is a modified version of the widely used ResNet-101 (He et al., 2016) that incorporates the concept of “deep supervision” to apply intermediate supervision for guiding the training process. This approach involves adding auxiliary classifiers to the intermediate layers of the network, which facilitates the flow of gradients and helps in better convergence during training. In comparison to the ResNet-101, the ResNet-101-D architecture introduces a modification to the ResNet-101 architecture by incorporating a 2 × 2 average pooling layer with a stride of 2 before the convolutional layers. This modification results in a larger receptive field, which enables the network to capture more contextual information and improve its ability to handle complex visual data.

**Figure 3:**
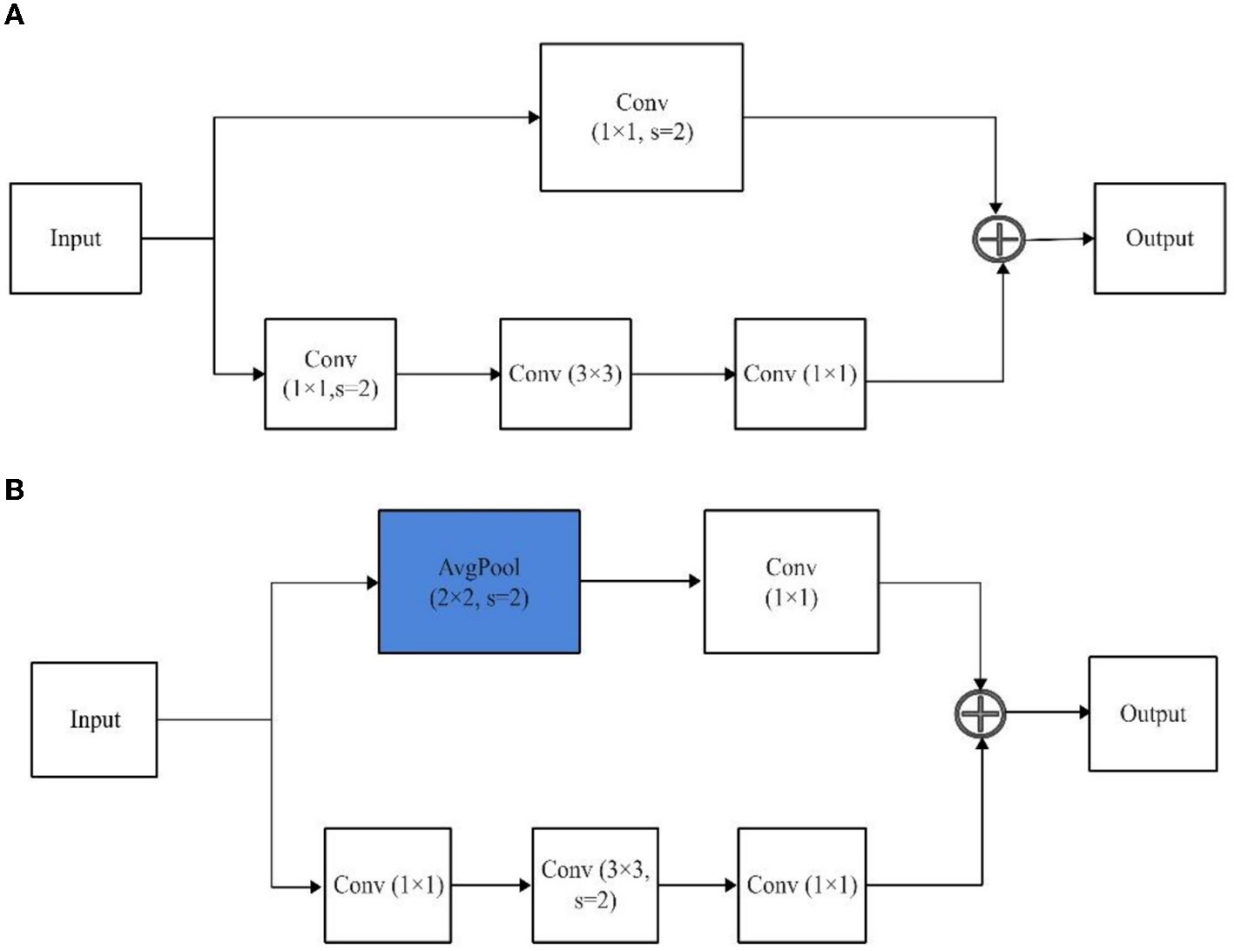
The architecture of a block of ResNet and ResNet-D. **(A)** ResNet. **(B)** ResNet-D.

In the context of cow weight prediction based on semantic segmentation, the ResNet-101-D has the potential to enhance segmentation accuracy by selectively emphasizing informative features and suppressing less informative ones. This approach can lead to improved boundary localization of cow instances in images, ultimately contributing to enhanced weight prediction accuracy. As illustrated in Figure 3, the introduction of a 2 × 2 average pooling layer with a stride of 2 before the convolutional layers in the block of ResNet-D leads to a downsampling of the input feature maps by a factor of 2, effectively reducing their spatial dimensions. This downsampling process expands the receptive field of subsequent convolutional layers, enabling them to capture a greater extent of global contextual information. Additionally, the downsampling operation reduces the computational cost of subsequent convolutional layers by reducing the number of input feature maps. The pooling layer also aids in decreasing the number of parameters in subsequent convolutional layers, potentially preventing overfitting, and enhancing generalization performance.

#### 2.2.2 ASPP-SE

To achieve accurate weight prediction based on visual features, it is essential to capture global contextual information and segment cow instances of varying sizes within images. The ASPP module is a convolutional neural network component that enables the network to capture multi-scale contextual information, making it highly effective for object detection and segmentation (Chen et al., 2017; Ding et al., 2023). It utilizes multiple parallel atrous convolutions with different dilation rates to capture features at various spatial resolutions, enabling the network to identify objects of different sizes. However, in the complex agricultural settings, it may not always capture the most informative features relevant to cow weight prediction. To address this limitation, a combination of the ASPP module with the Squeeze-and-Excitation (SE) module is proposed to enhance the accuracy of the semantic segmentation model for cow weight prediction (Hu et al., 2018), which emphasizes informative features while suppressing less relevant ones through feature recalibration using a gating mechanism. These informative features encompass visual cues and patterns directly correlated with cow weight, such as size, posture, or specific anatomical features of the cow within the images.

The incorporation of the SE mechanism into the ASPP module allows for the selective emphasis of the most informative features of the extracted features through a channel-wise weighting scheme. This results in the mitigation of the impact of irrelevant or noisy features that may not be associated with cow weight. The SE mechanism achieves this by reducing the spatial dimensions of the feature maps to 1 × 1 using a global average pooling operation, followed by a squeeze operation that decreases the dimensionality of the feature maps in the channel dimension. The excitation operation then selectively amplifies the informative features while suppressing the less informative ones. The resulting sigmoid activation function produces a channel-wise weighting mask that highlights the informative features of the input feature maps.

#### 2.2.3 Decoder and segmentation head

The decoder module begins by performing an upsampling operation, which increases the size of the feature map to match that of the input image. Deconvolution, also known as a deconvolution layer, is employed as the upsampling technique. Deconvolution achieves upsampling by applying a convolution operation to the feature map, effectively enlarging its dimensions. The deconvolution layer incorporates learnable parameters that adapt the feature transformation during upsampling by learning specific convolutional kernel weights.

Following the upsampling process, the decoder's output is fused with the low-level features from the backbone network. This fusion operation aims to combine contextual information with the lower-level features. To maximize the utilization of the low-level features within the encoder, skip connections are introduced within the decoder. These skip connections connect the corresponding level feature maps from the encoder to the corresponding level feature maps in the decoder. By establishing these connections, the decoder can integrate the low-level feature information with the high-level contextual information, ultimately enhancing the accuracy of the semantic segmentation process.

The segmentation head serves as the final layer of the model and is responsible for transforming the feature map generated by the decoder into the ultimate semantic segmentation prediction. It consists of a convolutional layer and a pixel classifier. The convolutional layer performs crucial adjustments to the number of channels or the resolution of the feature map to meet the specific requirements of the semantic segmentation task. This adaptation enables the model to effectively extract informative features and capture contextual information from the image data. On the other hand, the pixel classifier assigns each pixel to its corresponding semantic category, thus achieving pixel-wise semantic segmentation.

### 2.3 Weight prediction based on regression-based machine learning methods

In this study, a combination of five indicators was employed to predict cow weight, namely the areas of top-view and back-view of the cows, the height of the top-view shooting distance from the cow, back-view shooting distance from cattle, and the cow's age. The areas of first two views were obtained by the proposed segmentation algorithm applied to the images of cows captured from various angles. These indicators served as input with corresponding weight as the target output, to train regression-based machine learning methods for weight prediction. Specifically, BP neural network, Support Vector Machine (SVM), Decision Tree (DT), Multiple Linear Regression (MLR), and Gaussian Regression (GR) were compared using various evaluation metrics to assess their performance.

BP neural network is a popular type of feedforward artificial neural network that utilizes the backpropagation algorithm to update the weights by minimizing the error between actual and predicted outputs during supervised training (Rumelhart et al., 1986). This approach enables the network to model complex non-linear relationships and make accurate predictions. The regression model for a neural network can be represented as:


$$\begin{array}{l} Y=f\left(w_{1}x_{1}+w_{2}x_{2}+w_{3}x_{3}+w_{4}x_{4}+w_{5}x_{5}+b\right) \end{array}$$

Here, *Y* is the target variable (weight). *f* is the activation function, and *w*_1_, *w*_2_, *w*_3_, *w*_4_, and *w*_5_ are the weights. *x*_1_, *x*_2_, *x*_3_, *x*_4_, and *x*_5_are the input features, and b is the bias.

SVM is a widely used supervised learning algorithm in classification and regression tasks (Boser et al., 1992). It aims to identify an optimal hyperplane that maximally separates data points of different classes or predicts target values with the largest margin while minimizing the prediction errors. SVM is a powerful and versatile algorithm, capable of handling non-linearly separable data through the kernel trick. The goal of SVM regression is to find a function that minimizes the difference between predicted and actual values. The regression function can be expressed as:


$$\begin{array}{l} Y=\overset{n}{\underset{i}{\sum }}\alpha_{i}K\left(X_{i},X\right)+c \end{array}$$

Here, α_*i*_ represents the coefficients of support vectors. Each support vector has a corresponding coefficient, indicating the importance of that support vector in the model. *K*(*X*_*i*_, *X*) is the kernel function, and this function measures the similarity between the input sample *X* and the support vector *X*_*i*_ from the training data. *c* is the bias and represents the average deviation between the predicted and actual values.

DT is a hierarchical model utilized for classification and regression tasks (Quinlan, 1986). It segments the data space iteratively based on feature values, producing a tree-like structure where decision points are represented by nodes and leaf nodes signify the predicted outcome. DTs offer interpretability and ease of visualization.

MLR is a widely used statistical method for modeling the relationship between a dependent variable and multiple independent variables (Freund et al., 2006). It assumes a linear relationship between the variables and estimates the coefficients for each independent variable by minimizing the residual sum of squares. The method is highly interpretable and can provide insights into the relationships between variables. The regression equation for MLR takes the form:


$$\begin{array}{l} Y=\beta_{0}+\beta_{1}Z_{1}+\beta_{2}xZ_{2}+\beta_{3}xZ_{3}+\beta_{4}xZ_{4}+\beta_{5}Z_{5} \end{array}$$

Here, *Z*_1_, *Z*_2_, *Z*_3_, *Z*_4_, and *Z*_5_are the predictor variables and β_1_, β_2_, β_3_, β_4_, and β_5_ are the regression coefficients.

GR is also referred to as Gaussian Process Regression (Goldberg et al., 1997), is a non-parametric and probabilistic technique for modeling and predicting complex relationships. This method assumes a prior distribution over functions and updates it with observed data to obtain a posterior distribution. The resulting posterior distribution provides a probabilistic prediction of the output variable given the input data. GR is known for its flexibility in modeling various types of relationships, which makes it a popular choice in many applications.

### 2.4 Evaluation metrics

To assess the effectiveness of the proposed semantic segmentation method for extracting cattle body parameters, various commonly used evaluation metrics are utilized. The evaluation metrics used in this study are Intersection over Union (IoU), Accuracy, Frames Per Second (FPS), Average FPS (aFPS), Mean Intersection over Union (mIoU), and Mean Accuracy (mAcc). IoU measures the degree of overlap between the predicted mask and the ground truth mask. On the other hand, mIoU calculates the average degree of overlap between the predicted and ground truth masks. These metrics are frequently used to measure the accuracy of segmentation methods (He et al., 2021; Xu et al., 2021; Sheu et al., 2022). Accuracy is a measure of the proportion of correctly predicted pixels to the total number of pixels in the image. Meanwhile, mAcc measures the mean pixel-wise accuracy over the testing dataset. These metrics provide additional information on pixel-wise segmentation performance. FPS and aFPS are important for real-time applications since they measure the number of frames processed per second and the average FPS over the entire testing dataset respectively. These metrics are essential for determining the efficiency and practicality of the proposed semantic segmentation method.


$$\begin{array}{l} IoU=\frac{\text{Area of Intersection}}{\text{Area of Union}} \end{array}$$

$$\begin{array}{c} Accuracy\\ =\frac{\text{True Positive }+\text{ True Negative}}{\text{True Positive }+\text{ True Negative }+\text{False Positive }+\text{ False Negative }} \end{array}$$

$$\begin{array}{l} FPS=\frac{1}{\text{Time Per Frame}} \end{array}$$

$$\begin{array}{l} aFPS=\frac{\text{Total number of frames}}{\text{Total Time}} \end{array}$$

$$mIoU=\frac{\sum \text{IoU for each class}}{\text{Number of classes}}$$

$$m\text{Acc=}\frac{\sum \text{Accuracy for each class}}{\text{Number of classes}}$$

For regression-based methods analysis for weight prediction, a range of metrics are used to assess the model's overall accuracy and fit to the data. In this study, four widely used metrics are employed including root mean square error (RMSE), mean squared error (MSE), mean absolute error (MAE), and coefficient of determination (*R*-squared) (Chicco et al., 2021; Algarni and Ismail, 2023; Bansal and Singh, 2023). These metrics are computed using the following equations.


$$R^{2}=1-\frac{\sum {\left(y_{i}-{\hat{y}}_{i}\left(A_{t},\;A_{b},\;H_{t},\;H_{b},\;Age\right)\right)}^{2}}{\sum {\left(y_{i}-y_{mean}\right)}^{2}}$$

$$RMSE=\sqrt{\frac{\sum {\left(y_{i}-{\hat{y}}_{i}\left(A_{t},\;A_{b},\;H_{t},\;H_{b},\;Age\right)\right)}^{2}}{n}}$$

$$MSE=\frac{\sum {\left(y_{i}-{\hat{y}}_{i}\left(A_{t},\;A_{b},\;H_{t},\;H_{b},\;Age\right)\right)}^{2}}{n}$$

$$\begin{array}{l} MAE=\;\frac{\sum |y_{i}-{ŷ}_{i}\left(A_{t},\;A_{b},\;H_{t},\;H_{b},\;Age\right)|}{n} \end{array}$$

where *A*_*t*_ and *A*_*b*_ represent the areas of the top-view and back-view of the cow, *H*_*t*_ and *H*_*b*_ are the heights of the top-view and back-view shooting distances, and Age denotes the age of the cow *A*_*t*_.

## 3 Results and discussion

### 3.1 Semantic segmentation performance analysis

Accurate extraction of cattle body size parameters is crucial for reliable weight prediction, and this heavily relies on the performance of the semantic segmentation model. In this study, the proposed model was comprehensively evaluated, and the results were compared with those obtained from leading semantic segmentation algorithms. The main objective is to highlight the potential of the proposed model as a reliable and robust tool for extracting the body size parameters of cattle within complex environments, which can be used to enhance the accuracy of weight prediction.

**Figure 4:**
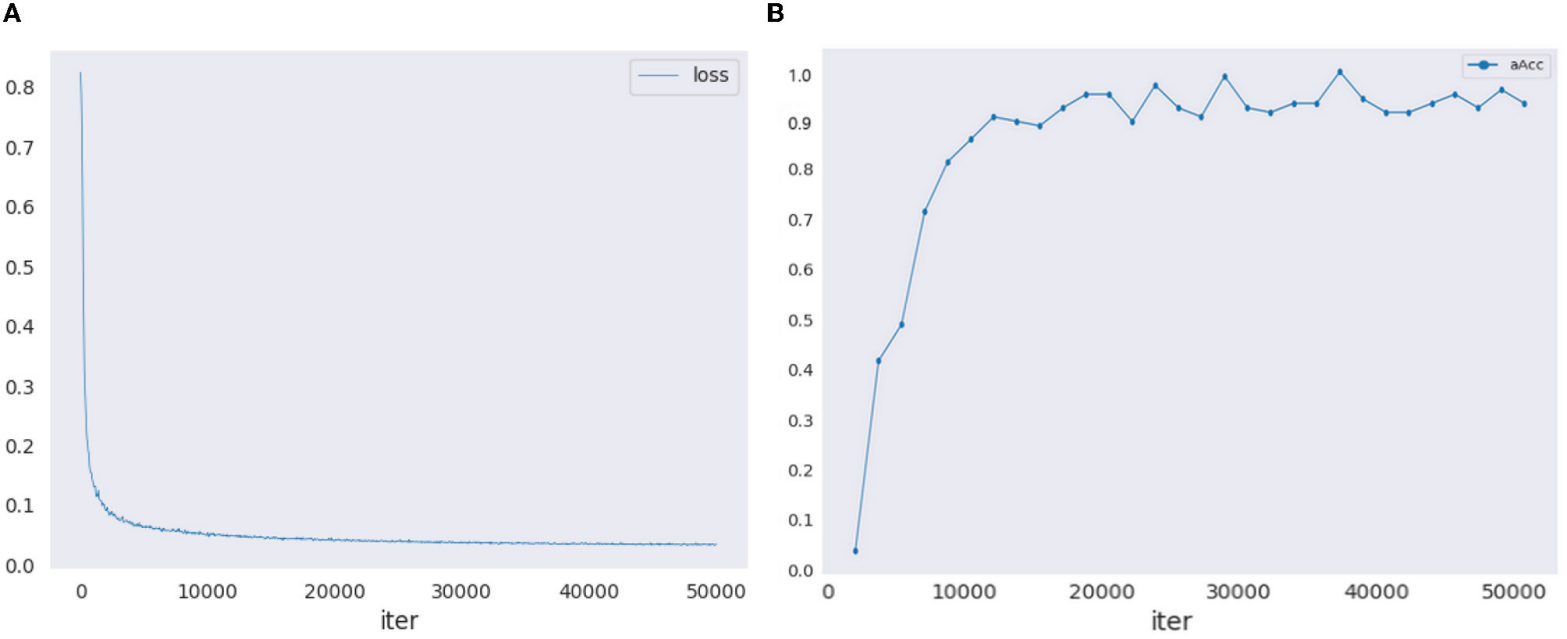
The training loss curve and validation accuracy curve. **(A)** Training loss curve. **(B)** Validation accuracy curve.

The training of the proposed model involves a meticulous selection of parameters to achieve optimal segmentation performance. A batch size of 8 was employed to balance memory constraints and computational efficiency during training. The initial learning rate was set to 0.001, implementing the 'poly' learning rate policy to dynamically adjust the learning rate based on the epoch for improved convergence. The model was trained for 50,000 iterations, ensuring sufficient iterations for the network to learn meaningful representations. Figure 4 illustrates the loss curve and validation accuracy curve during the training process. To augment the dataset and enhance the model's robustness, random horizontal flipping and random scaling were applied during training. Additionally, weight decay of 0.0005 was employed to regularize the model and prevent overfitting. The choice of these parameters was determined through empirical experimentation to strike a balance between model generalization and computational efficiency. The comparison experiments were conducted using the uniform parameter settings, encompassing both batch size and image augmentation techniques. Moreover, all experiments underwent optimization to achieve their peak performance.

#### 3.1.1 Evaluation of feature extraction network

To assess the suitability of the backbone network used in this study, a thorough comparison was conducted among four different ResNet architectures, namely ResNet 50, ResNet 101, ResNet-50-D, and ResNet-101-D. The purpose of this analysis was to determine which ResNet architecture would provide the best semantic segmentation results for the cattle body size parameter extraction task.

The findings presented in Table 1 indicate that ResNet-101-D outperforms the other architectures across all the evaluated metrics, with a 0.1% increase in mAcc and a 0.3% increase in mIoU, compared to the second-best performing architecture, ResNet-50-D. Although ResNet-101-D exhibited slightly lower FPS and aFPS values, the performance gains in other metrics justify this trade-off. While ResNet 50 had the highest FPS and aFPS, it achieved the lowest accuracy and IoU among the four architectures. ResNet 101 performed well and had scores similar to ResNet-101-D, but with slightly lower scores in IoU, mIoU, and mAcc. These results suggest that the ResNet_vd architectures, which replace the 7 × 7 convolution in the input stem with three 3 × 3 convolutions and add a 2 × 2 avg_pool with stride 2 before the convolution in the downsampling block, are effective at improving semantic segmentation results. The results underscore that ResNet-101-D is capable of capturing a wider range of distinct features, leading to highly accurate and reliable semantic segmentation results for the task of cattle body size parameter extraction.

**Table 1:** Performance comparisons of different ResNet networks.

	**IoU**		**Accuracy**		**FPS**		**aFPS**	**mIoU**	**mAcc**
	**Cattleback**	**Cattlebody**	**Cattleback**	**Cattlebody**	**Cattleback**	**Cattlebody**			
ResNet 50	0.936	0.958	0.966	0.981	12.3	4.9	8.6	0.947	0.974
ResNet 101	0.937	0.959	0.974	0.982	9.2	3.9	6.6	0.948	0.978
ResNet-50-D	0.941	0.960	0.972	0.983	12.2	4.9	8.6	0.951	0.978
ResNet-101-D	0.946	0.962	0.974	0.984	9.1	3.9	6.5	0.954	0.979

Numerous studies have explored the performance of the ResNet-101-D model in comparison to other architectures across a wide range of tasks. For instance, ResNet-101-D has demonstrated superior performance in image classification (Kang et al., 2021), object detection (Deep learning based UAV type classification), and semantic segmentation tasks (Wu et al., 2020). It is worth noting that the trade-off between performance and speed should be carefully considered, depending on the specific application of the semantic segmentation task. In this work, ResNet-101-D appears to be the most suitable architecture for segmenting cattle body size parameters, considering its superior performance in IoU and accuracy, which are critical metrics for semantic segmentation. However, ResNet-50-D and ResNet 50 can also provide high performance and may be more suitable for tasks that require higher FPS and aFPS values. For example, in an intelligent spraying system, real-time processing is critical for ensuring timely and accurate detection and tracking of fruit disease (Storey et al., 2022).

#### 3.1.2 Ablation study of the attention mechanism

The inclusion of attention mechanisms has been shown to improve the segmentation performance of deep neural networks by enabling them to selectively focus on prominent regions while suppressing irrelevant information (Wang and He, 2022). This study compares three commonly used attention mechanisms namely, SE, Efficient Channel Attention (ECA) (Wang et al., 2020b), and Convolutional Block Attention Module (CBAM) (Woo et al., 2018) on the dataset used in the experiment. Compared to the SE module utilized in this paper, the ECA module aims to enhance inter-channel correlations for more effectively capturing information across channels. It achieves this by introducing a lightweight 1D convolution operation, computing channel weights position-wise to reflect the inter-channel correlations. On the other hand, the CBAM module takes a holistic approach by considering both channel and spatial dimensions of attention. It comprises two components: a channel attention module and a spatial attention module. The channel attention is implemented through the SE module, while spatial attention weights different spatial positions by leveraging inter-channel correlations.

The analysis of Table 2 indicates that the SE attention mechanism surpasses other attention mechanisms, such as ECA and CBAM, in enhancing semantic segmentation performance. The SE mechanism produced significant improvements in critical metrics, including IoU and accuracy, achieving the highest values for both cattle back (0.946) and cattle body (0.962), as well as 0.974 and 0.984 accuracy values for cattle back and cattle body, respectively. These enhancements represent a 0.5% and 0.6% increase in mIoU and mAcc compared to the model without attention. In contrast, both ECA and CBAM exhibited performance improvements relative to the model without attention but fell short in overall performance compared to the SE mechanism. This indicated that the channel and spatial attention mechanisms employed by ECA and CBAM were not as effective in capturing the most relevant features in the context of semantic segmentation, particularly for cattle body size parameter extraction.

**Table 2:** Performance comparisons of three attention mechanisms.

	**IoU**		**Accuracy**		**FPS**		**aFPS**	**mIoU**	**mAcc**
	**Cattleback**	**Cattlebody**	**Cattleback**	**Cattlebody**	**Cattleback**	**Cattlebody**			
None	0.939	0.953	0.970	0.975	10.3	4.6	7.5	0.946	0.973
SE	0.946	0.962	0.974	0.984	9.1	3.9	6.5	0.954	0.979
ECA	0.941	0.955	0.971	0.978	9.4	3.5	6.5	0.948	0.975
CBAM	0.944	0.959	0.973	0.980	9.3	3.4	6.4	0.952	0.977

The observed discrepancies in performance metrics among the attention mechanisms can be ascribed to their distinct underlying structural characteristics. The superior performance of the SE mechanism can be attributed to its ability to capture critical features effectively, resulting in higher IoU and accuracy values for both cattle back and cattle body. However, the SE mechanism had slightly lower FPS (9.1) and aFPS (6.5) values than the attention-less model. Nonetheless, the trade-off between improved segmentation performance and slightly lower FPS and aFPS values was considered acceptable. This can be explained by the increased computational complexity introduced by the SE mechanism, as it learns to focus on essential features in the input data. Therefore, the SE attention mechanism in this work was selected as the most favorable choice for the semantic segmentation model under investigation, providing more accurate and reliable results.

#### 3.1.3 Comparisons with typical algorithms

To further validate the advanced capabilities of the algorithm proposed in this study, a comparative analysis was performed against state-of-the-art algorithms commonly used in the field, namely PSPNet (Zhao et al., 2017), PSANet (Zhao et al., 2018), OCRNET (Yuan et al., 2020), and HRNET (Wang et al., 2020a). These algorithms were chosen due to their prominent standing in the field and their demonstrated efficacy in similar tasks including fruits segmentation (Qiao et al., 2022; Qi et al., 2022), land segmentation (Yuan et al., 2021), crop and weed segmentation (Huang et al., 2021; Yang et al., 2023).

A thorough analysis of Table 3 highlights the superiority of the proposed method in accurately extracting cattle body size parameters compared to other four well-established algorithms. The proposed method achieved the highest IoU values for both cattle back (0.946) and cattle body (0.962), indicating its exceptional segmentation performance. Moreover, it attained the top accuracy values of 0.974 and 0.983 for cattle back and cattle body respectively, which outperformed the second-best algorithm, PSPNet, by 0.5% and 0.2% in IoU, and 0.4% and 0.2% in accuracy. Furthermore, the proposed method excels in terms of mIoU (0.954) and mAcc (0.979), emphasizing its overall effectiveness in the cow body segmentation tasks. Although the FPS (9.1) and aFPS (6.5) values of the proposed method are slightly lower than some of the other algorithms, the superior performance in segmentation quality compensates for the reduced frame rates. This trade-off signifies the model's ability to balance computational efficiency with highly accurate cattle body size parameter extraction.

**Table 3:** Performance comparisons with typical algorithms.

	**IoU**		**Accuracy**		**FPS**		**aFPS**	**mIoU**	**mAcc**
	**Cattleback**	**Cattlebody**	**Cattleback**	**Cattlebody**	**Cattleback**	**Cattlebody**			
PSPNet	0.941	0.960	0.970	0.981	9.8	5.0	7.4	0.951	0.976
PSANet	0.940	0.959	0.973	0.981	8.7	4.4	6.6	0.950	0.977
OCRNET	0.936	0.960	0.972	0.980	11.5	8.1	9.8	0.948	0.976
HRNET	0.938	0.960	0.968	0.979	12.0	10.2	11.1	0.949	0.974
The proposed method	0.946	0.962	0.974	0.984	9.1	3.9	6.5	0.954	0.979

**Figure 5:**
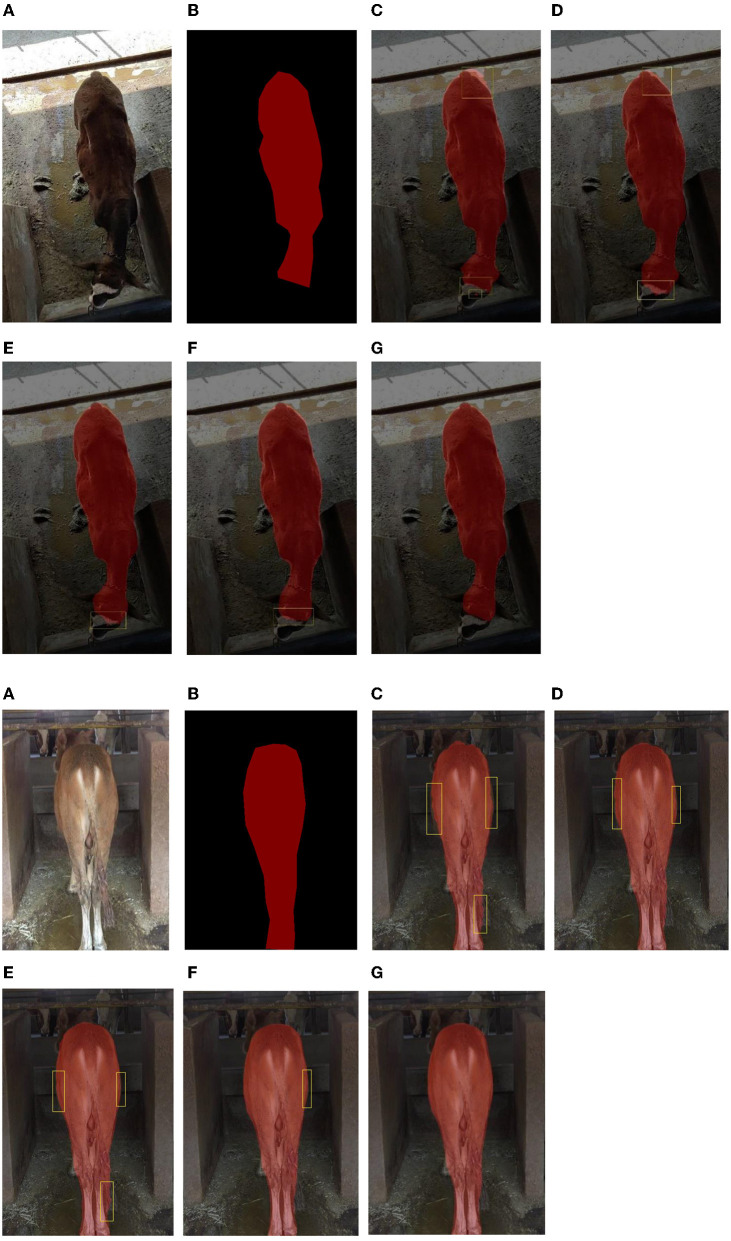
Comparisons of segmentation results using various models. **(A)** Image. **(B)** Ground truth. **(C)** HRNET. **(D)** OCRNET. **(E)** PSPNet. **(F)** PSANet. **(G)** The proposed method.

The proposed algorithm exhibited robustness and generalizability in practical and challenging scenarios, as evidenced by its consistent performance and adaptability. In contrast, the competing algorithms showed varying degrees of limitations in handling complex and challenging instances, resulting in decreased performance particularly in cases involving complex instances, as shown in areas marked with yellow rectangles in Figure 5. Such instances may involve intricate object shapes, occlusions, or challenging backgrounds. The proposed algorithm in this study was designed to address the specific requirements of practical applications for obtaining the cattle body size parameters, making it a highly promising choice for real-world implementations. Therefore, it is crucial to subject the latest algorithms to extensive testing and validation in various practical scenarios prior to their application to ensure its effectiveness and applicability.

### 3.2 Regression-based weight prediction analysis

This work conducted an investigation into the performance of MLR, DT, GR, SVM, and BP neural network models for predicting cattle weights using a combination of image-derived cattle body area parameters, individual age and shooting distances as input variables. The models were trained and evaluated using a dataset consisting of actual cattle weights as the target variable and the aforementioned input variables.

This analysis aimed to evaluate the performance of the models in estimating weight values based on the provided input features and assess its potential for practical applications in weight prediction. The coefficient of determination, *R*^2^, serves as a measure of the goodness of fit of the regression models. It indicates the proportion of variance in the predicted cattle weights that can be explained by the input variables. Higher *R*^2^ values signify a stronger correlation and a better fit between the predicted and actual weights.

As presented in Table 4, various evaluation metrics were calculated to assess the performance of different regression models based on the predicted and actual weights of the training samples. The results of the analysis indicated that the BP neural network model achieved the highest goodness of fit among the evaluated regression models for predicting individual beef cattle weights. With an *R*^2^ value of 0.99, the BP neural network model exhibited a strong correlation between the predicted and actual weights. Furthermore, it demonstrated the lowest MAE of 18.48 pounds and RMSE of 22.00 pounds, indicating its superior accuracy and precision in weight prediction.

**Table 4:** Error analysis of different weight prediction models on training samples.

**Model**	**R^2^**	**RMSE**	**MSE**	**MAE**
MLR	0.98	35.01	1,225.72	28.44
DT	0.95	53.31	2,841.80	37.09
SVM	0.98	35.67	1,272.42	28.19
GR	0.97	40.87	1,670.18	31.59
BP	0.99	22.00	483.85	18.48

Following the BP neural network, both the MLR and SVM models exhibited favorable performance. These models achieved *R*^2^ values of 0.98 and MAEs of 28.44 pounds and 28.19 pounds, respectively. The corresponding RMSE values were 35.01 pounds and 35.67 pounds, respectively. These results suggest a good fit between the predicted and actual weights, albeit slightly less accurate compared to the BP neural network model. The Gaussian process regression model demonstrated relatively lower performance compared to the aforementioned models, yielding an *R*^2^ value of 0.97, an MAE of 31.59 pounds and an RMSE of 40.87 pounds. Although it exhibited a weaker fit, the model still provided reasonable predictions of individual cattle weights. The DT regression model presented the lowest goodness of fit among the evaluated models, with an *R*^2^ value of 0.95. It also presented a higher MAE of 37.09 pounds and a larger RMSE of 53.31 pounds, indicating less accurate predictions compared to the other models.

**Figure 6:**
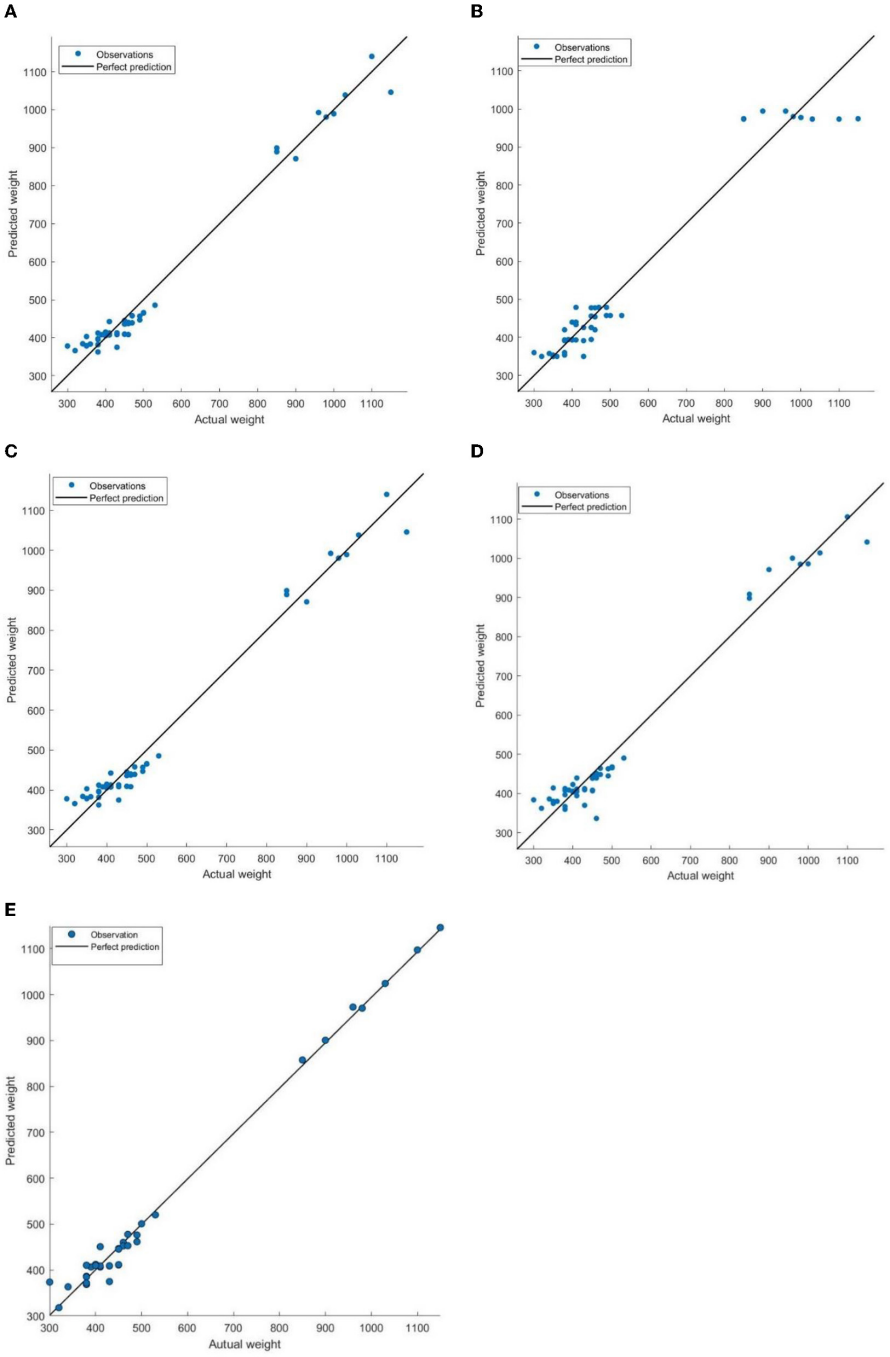
Comparisons of predicted weight and actual weight (pounds) on training samples. The ideal predicted values, where the predicted weight equals the actual weight, are denoted by a black solid line. The actual weights are represented by blue dots. **(A)** MLR. **(B)** DT. **(C)** SVM. **(D)** GR. **(E)** BP.

Figure 6 presents the fit between predicted and actual weights for regression models using training samples. The analysis revealed that the BP neural network produced predictions that closely aligned with the actual weights, as evidenced by the actual weights clustering around the ideal predicted values. This observation signified a strong fit between the predicted and actual weights. In addition, both the SVM and MLR models exhibited similar patterns. The majority of actual weight values clustered around the predicted values, with only one or two outliers deviating from the expected trend. In contrast, the GR and DT models exhibited larger errors overall, particularly when predicting weights exceeding 800 pounds. This instability highlights the limitations of these models in accurately predicting individual cattle weights.

To further validate the prediction performance of the BP neural network model, this study employed testing samples to evaluate regression models for comparisons, as illustrated in Figure 6 and Table 5. The findings in Figure 6 indicate that both the SVM and BP neural network models exhibited superior prediction results on the testing samples. Table 5 further revealed that these two models yield smaller MAEs and RMSEs compared to the other models. This can be attributed to their robust nonlinear mapping capabilities, which enable them to capture complex nonlinear relationships and effectively reflect the fuzzy relationship between various indicators and weight, resulting in enhanced adaptability compared to linear and other nonlinear models. This advantage alleviates the need for excessive concern about collinearity issues among indicators, ultimately leading to higher prediction accuracy.

**Table 5:** Error analysis of different weight prediction models on testing samples.

**Model**	**R^2^**	**RMSE**	**MSE**	**MAE**
MLR	0.97	45.69	2,087.58	34.39
DT	0.89	60.24	3,628.86	39.75
SVM	0.97	29.86	891.62	28.97
GR	0.93	52.31	2,736.34	34.58
BP	0.98	22.73	516.59	13.11

However, it is worth noting that the optimization objective of the BP neural network model is based on minimizing empirical risk, which may lead to potential convergence to local optima during training, thus resulting in less stable test results. Therefore, further validation with an increased sample size would be beneficial. On the other hand, SVM regression follows the principle of structural risk minimization, ensuring better generalization ability of the model. The model's small sample learning approach and convergence to the global optimum contribute to its superior performance on the test samples compared to the training samples.

Consistent with the findings obtained from the training samples, the prediction performance of the testing samples followed a similar pattern. However, the performance of MLR, GR, and DT models was slightly degraded on the testing samples compared to the training samples. The maximum absolute error observed in the test samples was higher than that in the training samples, which could be attributed to the smaller size of the testing sample or inadequate generalization ability of the models.

**Figure 7:**
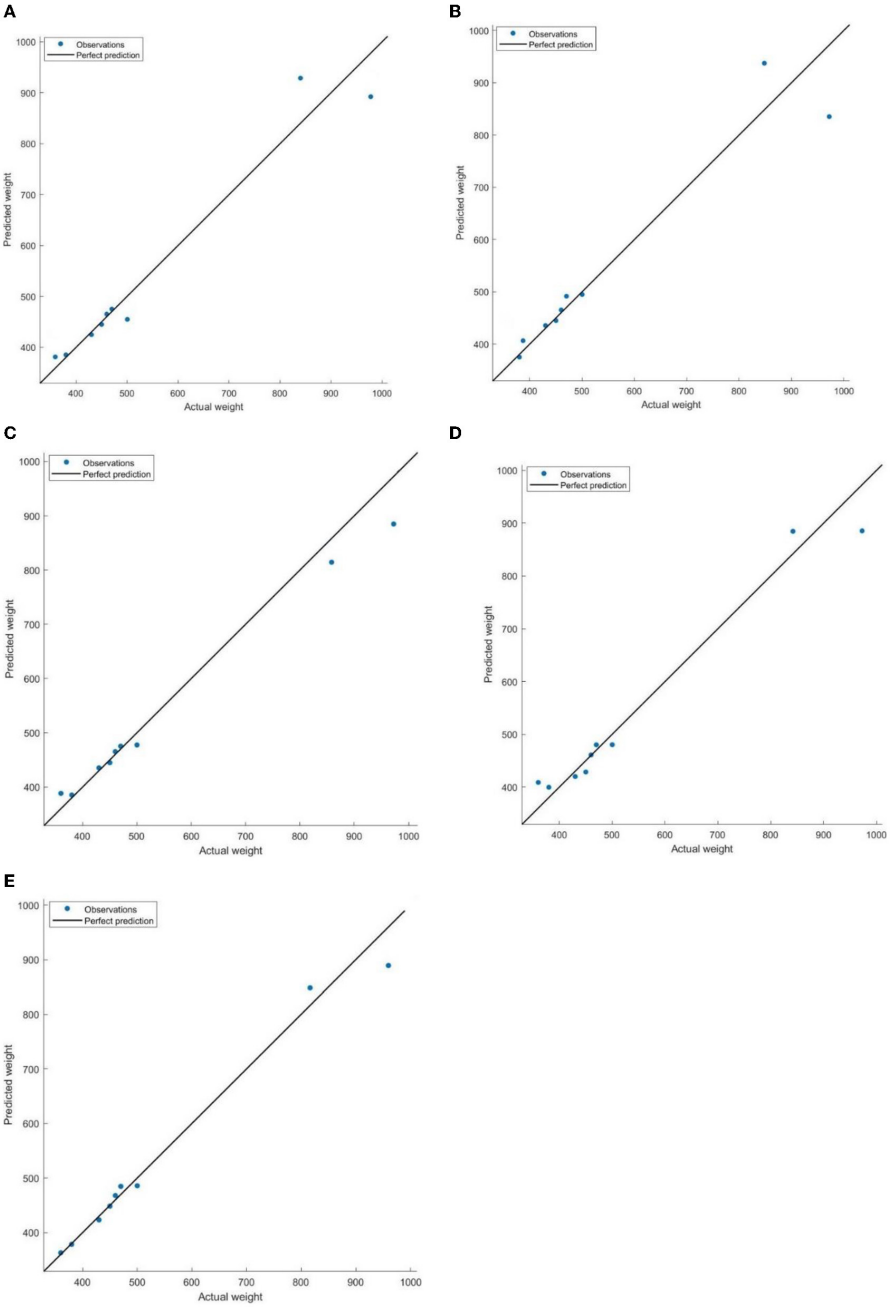
Comparisons of predicted weight and actual weight (pounds) on testing samples. The perfect prediction is represented by the alignment of the black solid line and the blue dots on the graph, indicating an accurate prediction where the model precisely estimates the weights. **(A)** MLR. **(B)** DT. **(C)** SVM. **(D)** GR. **(E)** BP.

Additionally, Figure 7 illustrates that the absolute errors between predicted and actual weights for all five weight prediction models were more prominent when the individual weight of cattle exceeded 800 pounds. This can be explained by the decelerated growth rate that typically occurred after 12 months of age. It should be noted that the weight range of 800 pounds fell within the timeframe of 12–18 months, during which the growth rate of cattle tended to decrease. Consequently, the models may face challenges in accurately capturing the complex growth patterns and specific characteristics associated with this weight range. Additionally, the limited number of samples available above 800 pounds in this study further restricts the models' ability to fully learn and generalize the weight variations in this specific range. Considering the overall performance, the BP neural network model emerged as the preferred choice for predicting cattle weights.

The trained BP neural network utilized the Levenberg-Marquardt backpropagation algorithm to model the complex relationships within the dataset. The final regression model obtained consists of 10 hidden layer neurons. The weights and bias connecting each input variable to the hidden layer neurons are shown in Table 6. Each row in the weight matrix corresponds to a hidden layer neuron, and each column corresponds to a specific input variable. The positive and negative signs of the weights indicate the direction of influence, while the magnitude of the weights reflects the strength of that influence. Additionally, the bias parameters associated with each hidden layer neuron contribute to the overall predictive power of the neural network.

**Table 6:** BP neural network weight matrix and bias for feature variables.

	**Age**	**Top-view height**	**Top-view area**	**Back-view distance**	**Back-view area**	**Bias**
Neuron 1	−0.7168	1.7498	1.0712	0.2550	0.0099	2.2288
Neuron 2	1.5923	1.4301	−0.0613	−0.3326	−0.1688	1.9718
Neuron 3	−1.0202	1.1312	1.1350	−0.2815	0.2398	−1.1727
Neuron 4	1.4218	0.1317	1.2314	−1.2518	1.6771	0.4229
Neuron 5	0.3109	0.8227	0.1027	−1.3699	−1.2313	−0.0952
Neuron 6	−1.6136	−0.8355	1.2502	1.4542	−0.4391	0.4524
Neuron 7	−0.5799	0.2280	1.3392	1.4404	0.3207	−0.0802
Neuron 8	−0.8586	2.2939	−1.1279	−1.1543	0.3060	−1.1329
Neuron 9	1.0519	0.6161	0.3122	1.1347	1.0746	−2.2368
Neuron 10	0.8432	0.7209	−0.8869	−1.3034	0.4020	2.0389

To illustrate the impact of semantic segmentation method improvements on the final weight prediction, this study conducted a weight prediction analysis on the testing set using a segmentation method without the inclusion of an attention mechanism. The results are presented in Table 7. In contrast to the results presented in Table 5, the results suggest that the absence of an attention mechanism led to a decrease in the performance of all weight prediction models. Specifically, there is a noticeable decrease in *R*^2^ and an increase in RMSE, MSE, and MAE, emphasizing the beneficial impact of attention mechanisms on improving the accuracy of weight estimation. Furthermore, in alignment with the segmentation results that include attention mechanism, the BP neural network consistently exhibits superior performance in weight prediction accuracy compared to other models.

**Table 7:** Weight prediction error analysis on testing samples without attention mechanism.

**Model**	**R^2^**	**RMSE**	**MSE**	**MAE**
MLR	0.95	48.86	2,387.30	43.22
DT	0.88	63.08	3,979.60	56.59
SVM	0.96	32.41	1,050.41	30.47
GR	0.91	60.90	3,708.93	53.48
BP	0.96	25.32	641.10	18.75

## 4 Limitations and future work

This study presents a promising approach for predicting cattle weight based on semantic and BP neural network, however, there are several limitations that need to be addressed. These limitations provide valuable insights for future research and development in this field.

The first limitation pertains to the quality and uniformity of the image data used in the study. Despite employing advanced techniques such as the ResNet-101-D model with the SE mechanism for image processing, uncontrollable environmental factors, such as background clutter, could introduce noise into the data, potentially impacting the accuracy of the models. This finding aligns with previous research (Zhang et al., 2022), which discussed similar challenges in machine learning for animal recognition. Variations in lighting conditions and certain postures adopted by the animals, such as standing on their back feet, further exacerbated these issues. Additionally, accurate segmentation of the legs and head from back views proved challenging, which added complexity to the image analysis process. Moreover, as highlighted by Johnson and Smith in their work regarding the camera quality and its impact on image-based machine learning models, camera quality and stability can significantly influence the quality of the collected data, ultimately affecting the performance of the models. The use of automated measurement techniques or advanced imaging technologies, such as 3D scanning or infrared imaging, will improve the accuracy and reliability of the data.

A substantial limitation observed in the study is the decline in model performance with increasing cattle weight, particularly weights exceeding 800 pounds. This limitation stems from a relative scarcity of data in this weight range in the dataset, leading to an imbalance that hampers the model's learning efficacy. As discussed in the detrimental impact of data imbalance on model performance (Buda et al., 2018; Johnson and Khoshgoftaar, 2019), future studies should aim to collect a larger and more diverse dataset that encompasses a wide range of cattle breeds, ages, and geographic locations. This would improve the generalizability of the weight prediction model and enhance its applicability to different populations. The current limitation on the size of the testing dataset affects the robustness of the statistical inferences drawn from the regression model. To address this concern, future studies will prioritize the inclusion of a more extensive testing dataset to ensure the reliability and validity of the statistical analyses performed. A larger testing dataset would contribute to more robust statistical evaluations, increasing the confidence in the model's predictive performance and the overall findings of the regression analysis. Additionally, exploring the unique growth patterns and characteristics of cattle exceeding 800 pounds might necessitate distinct modeling approaches or features for accurate prediction. Future research endeavors will also consider incorporating statistical tests, such as *T*-tests, to evaluate the significance of observed differences in model performance. This additional statistical scrutiny would provide a more comprehensive understanding of the model's performance and contribute to the overall rigor of the study.

The optimization objective of the BP neural network model, based on empirical risk minimization, poses another limitation. Convergence toward local optima is a well-established challenge in the machine learning community, as noted by LeCun et al. (2015). This issue can impact the stability and generalizability of the predictions across diverse datasets or cattle populations. Exploring alternative optimization strategies or algorithms, such as stochastic gradient descent, Adam optimizer, or simulated annealing, could be a potential future direction to overcome the convergence challenges faced by the BP neural network model.

The reliance on static variables such as body area parameters, individual age, and shooting distances in the current study may not fully capture the dynamic nature of cattle weights. Factors such as dietary habits, seasonal changes, health conditions, and genetic predisposition, which are known to influence cattle weights, were not considered in the analysis. Therefore, it is important for future research to focus on integrating multiple sources of data to provide a more comprehensive understanding of the factors influencing cattle weight. By incorporating these additional modalities into the prediction model, the accuracy and precision of weight estimations can be enhanced. This integration of diverse data sources will enable a more holistic approach to livestock weight prediction and contribute to more accurate and reliable outcomes.

The images presented in the dataset predominantly feature single cows from an optimal viewing angle, which may not fully represent the challenges encountered in real-world cattle farm environments. The accurate detection of the proper view, such as a back-view, of a cow for weight estimation within images containing multiple cows remains a challenge. Future work should also focus on validating the model on a larger scale and conducting field tests in real-world farming environments. This validation process will provide an opportunity to evaluate the model's performance under diverse conditions and ascertain its effectiveness in practical scenarios. Collaborating with farmers and industry stakeholders is vital in this regard, as their expertise and feedback can offer valuable insights and ensure that the model meets the specific needs and requirements of the agricultural community.

The precision and dependability of segmentation outcomes are closely tied to the caliber of human-conducted labeling. In this study, the visual precision of the annotations in the ground truth may seem rudimentary and might not faithfully represent intricate details. The creation of the ground truth involves human judgment, thereby presenting the possibility of inaccuracies and predispositions. In future research endeavors, addressing the limitations associated with human-dependent ground truth annotation will be paramount. One potential avenue for improvement involves exploring automated or semi-automated annotation methods to enhance the accuracy and granularity of segmentation results.

## 5 Conclusion

Accurate and efficient measurement of animal weight plays a pivotal role in various aspects of livestock production, health monitoring, animal welfare and stress reduction. In this study, we presented a non-contact and intelligent weight prediction approach for cows using computer vision and machine learning techniques. This method utilized image analysis and machine learning algorithms to estimate the weight of cows by measuring their external morphological features. By leveraging semantic segmentation techniques, precise boundaries and outer contours were extracted from cow images, which were subsequently utilized to train a regression-based model for weight prediction.

The application of pixel-level segmentation using the ResNet-101-D model with the SE mechanism allowed for precise extraction of cattle body size parameters, which served as essential inputs for weight prediction. Extensive evaluation revealed that the ResNet-101-D model exhibited superior performance and demonstrated its suitability for the task at hand. The incorporation of the SE mechanism yielded significant improvements in both accuracy and IoU metrics. The adaptive recalibration enabled by the SE mechanism enhanced the model's ability to capture fine-grained details and accurately segment cattle bodies.

For weight prediction, the BP neural network model was selected due to its commendable performance. It demonstrated a high level of accuracy in predicting individual cattle weights, particularly within the available weight range. However, the model's performance showed a slight decline when predicting weights exceeding 800 pounds, which can be attributed to the limited amount of data in this weight range. It is postulated that the paucity of data for weights within this range induced an imbalance, consequently impacting the learning efficacy of the model. This is consistent with similar constraints observed in extant literature (Ou and Murphey, 2007; Yu et al., 2021). Future research should focus on acquiring more data and dynamic factors in this range to enhance the model's performance.

The findings of this study contribute to the field of computer vision and provide a valuable tool for accurate cattle weight prediction, enabling advancements in livestock management and agricultural practices. Future research can explore the generalizability of the proposed approach to other animal species and investigate its potential for integration into real-world applications.

## Data availability statement

The raw data supporting the conclusions of this article will be made available by the authors, without undue reservation.

## Ethics statement

The animal studies were approved by Jiangxi Academy of Agricultural Sciences, Institute of Animal Husbandry and Veterinary Laboratory, and Animal Ethics Committee. The studies were conducted in accordance with the local legislation and institutional requirements. Written informed consent was obtained from the owners for the participation of their animals in this study.

## Author contributions

BX: Conceptualization, Data curation, Formal analysis, Investigation, Methodology, Software, Validation, Visualization, Writing – original draft. YM: Formal analysis, Investigation, Methodology, Writing – original draft. WW: Conceptualization, Project administration, Supervision, Writing – review & editing. GC: Funding acquisition, Project administration, Resources, Supervision, Writing – review & editing.

## References

[B1] Alawneh J. Stevenson M. Williamson N. Lopez-Villalobos N. Otley T. (2011). Automatic recording of daily walkover liveweight of dairy cattle at pasture in the first 100 days in milk. J. Dairy Sci. 94, 4431–4440. 10.3168/jds.2010-4002 21854916

[B2] Algarni M. Ismail M. M. B. (2023). Applications of artificial intelligence for information diffusion prediction: regression-based key features models. Int. J. Adv. Comput. Sci. Appl. 14. 10.14569/IJACSA.2023.01410123

[B3] Ashfaq M. Razzaq A. Haq S. U. Muhammad G. (2015). Economic analysis of dairy animal diseases in Punjab: a case study of Faisalabad district. J. Anim. Plant Sci. 25, 1482–1495.

[B4] Bansal S. Singh G. (2023). “Multiple linear regression based analysis of weather data: assumptions and limitations,” in International Conference on Advanced Communication and Intelligent Systems (Warsaw). 10.1007/978-3-031-45121-8_19

[B5] Berckmans D. (2014). Precision livestock farming technologies for welfare management in intensive livestock systems. Rev. Sci. Tech. 33, 189–196. 10.20506/rst.33.1.2273 25000791

[B6] Berry D. Lee J. Macdonald K. Stafford K. Matthews L. Roche J.. (2007). Associations among body condition score, body weight, somatic cell count, and clinical mastitis in seasonally calving dairy cattle. J. Dairy Sci. 90, 637–648. 10.3168/jds.S0022-0302(07)71546-1 17235139

[B7] Blokhuis H. Jones R. Geers R. Miele M. Veissier I. (2003). Measuring and monitoring animal welfare: transparency in the food product quality chain. Anim. Welfare-Potters Bar Then Wheathampstead 12, 445–456. 10.1017/S096272860002604X

[B8] Borges Oliveira D. A. Ribeiro Pereira L. G. Bresolin T. Pontes Ferreira R. E. Reboucas Dorea J. R. (2021). A review of deep learning algorithms for computer vision systems in livestock. Livest. Sci. 253:104700. 10.1016/j.livsci.2021.104700

[B9] Boser B. E. Guyon I. M. Vapnik V. N. (1992). “A training algorithm for optimal margin classifiers,” in Proceedings of the Fifth Annual Workshop on Computational Learning Theory (New York, NY). 10.1145/130385.130401

[B10] Buda M. Maki A. Mazurowski M. A. (2018). A systematic study of the class imbalance problem in convolutional neural networks. Neural Netw. 106, 249–259. 10.1016/j.neunet.2018.07.011 30092410

[B11] Charmley E. Gowan T. Duynisveld J. (2006). Development of a remote method for the recording of cattle weights under field conditions. Aust. J. Exp. Agric. 46, 831–835. 10.1071/EA05314

[B12] Chen L.-C. Papandreou G. Schroff F. Adam H. (2017). Rethinking atrous convolution for semantic image segmentation. arXiv. [Preprint]. 10.48550/arXiv.1706.05587

[B13] Chicco D. Warrens M. J. Jurman G. (2021). The coefficient of determination R-squared is more informative than SMAPE, MAE, MAPE, MSE and RMSE in regression analysis evaluation. PeerJ Comput. Sci. 7:e623. 10.7717/peerj-cs.623 34307865 PMC8279135

[B14] Cominotte A. Fernandes A. Dorea J. Rosa G. Ladeira M. Van Cleef E.. (2020). Automated computer vision system to predict body weight and average daily gain in beef cattle during growing and finishing phases. Livest. Sci. 232:103904. 10.1016/j.livsci.2019.103904

[B15] Dang C. Choi T. Lee S. Lee S. Alam M. Park M.. (2022). Machine learning-based live weight estimation for hanwoo cow. Sustainability 14:12661. 10.3390/su141912661

[B16] Daros R. R. Eriksson H. K. Weary D. M. von Keyserlingk M. A. G. (2020). The relationship between transition period diseases and lameness, feeding time, and body condition during the dry period. J. Dairy Sci. 103, 649–665. 10.3168/jds.2019-16975 31704020

[B17] Ding P. Qian H. Zhou Y. Yan S. Feng S. Yu S.. (2023). Real-time efficient semantic segmentation network based on improved ASPP and parallel fusion module in complex scenes. J. of Real-Time Image Process. 20:41. 10.1007/s11554-023-01298-4

[B18] Dohmen R. Catal C. Liu Q. (2021). Image-based body mass prediction of heifers using deep neural networks. Biosyst. Eng. 204, 283–293. 10.1016/j.biosystemseng.2021.02.001

[B19] Dohmen R. Catal C. Liu Q. (2022). Computer vision-based weight estimation of livestock: a systematic literature review. New Zealand J. Agric. Res. 65, 227–247. 10.1080/00288233.2021.1876107

[B20] Du A. Guo H. Lu J. Su Y. Ruchay A. Pezzuolo A.. (2021). “Automatic heart girth measurement for cattle based on deep learning,” in 2021 IEEE International Workshop on Metrology for Agriculture and Forestry (MetroAgriFor) (Trento-Bolzano: IEEE). 10.1109/MetroAgriFor52389.2021.9628696

[B21] Duan E. Hao H. Zhao S. Wang H. Bai Z. (2023). Estimating body weight in captive rabbits based on improved mask RCNN. Agriculture 13:791. 10.3390/agriculture13040791

[B22] Freund R. J. Wilson W. J. Sa P. (2006). Regression Analysis. Amsterdam: Elsevier.

[B23] Gjergji M. Weber V. M. Silva L. O. C. Gomes R. C. Araújo T. L. A. C. Pistori H.. (2020). “Deep learning techniques for beef cattle body weight prediction,” in 2020 International Joint Conference on Neural Networks (IJCNN) (Glasgow: IEEE). 10.1109/IJCNN48605.2020.9207624

[B24] Goldberg P. Williams C. Bishop C. (1997). Regression with input-dependent noise: a Gaussian process treatment. Adv. Neural Inf. Process. Syst. 10, 493–499..

[B25] González L. A. Bishop-Hurley G. J. Handcock R. N. Crossman C. (2015). Behavioral classification of data from collars containing motion sensors in grazing cattle. Comput. Electron. Agricu. 110, 91–102. 10.1016/j.compag.2014.10.018

[B26] Gu J. Q. Wang Z. H. Gao R. H. Wu H. R. (2017). Cow behavior recognition based on image analysis and activities. Int. J. Agric. Biol. Eng. 10, 165–174. 10.3965/j.ijabe.20171003.3080

[B27] Hakem M. Boulouard Z. Kissi M. (2022). Classification of body weight in beef cattle via machine learning methods: a review. Procedia Comput. Sci. 198, 263–268. 10.1016/j.procs.2021.12.238

[B28] He K. Zhang X. Ren S. Sun J. (2016). “Deep residual learning for image recognition,” in Proceedings of the IEEE Conference on Computer Vision and Pattern Recognition (Las Vegas, NV: IEEE). 10.1109/CVPR.2016.90

[B29] He T. Zhang Z. Zhang H. Zhang Z. Xie J. Li M.. (2019). “Bag of tricks for image classification with convolutional neural networks,” in Proceedings of the IEEE/CVF Conference on Computer Vision and Pattern Recognition (Long Beach, CA: IEEE). 10.1109/CVPR.2019.00065

[B30] He Y. Yu H. Liu X. Yang Z. Sun W. Wang Y.. (2021). Deep learning based 3D segmentation: a survey. arXiv. [Preprint]. 10.48550/arXiv.2103.05423

[B31] Hou Z. Huang L. Zhang Q. Miao Y. (2023). Body weight estimation of beef cattle with 3D deep learning model: PointNet++. Comput. Electron. Agric. 213:108184. 10.1016/j.compag.2023.108184

[B32] Hu J. Shen L. Sun G. (2018). “Squeeze-and-excitation networks,” in Proceedings of the IEEE Conference on Computer Vision and Pattern Recognition (Salt Lake City, UT: IEEE). 10.1109/CVPR.2018.00745

[B33] Huang S. Han W. Chen H. Li G. Tang J. (2021). Recognizing zucchinis intercropped with sunflowers in UAV visible images using an improved method based on OCRNet. Remote Sens. 13:2706. 10.3390/rs13142706

[B34] Johnson J. M. Khoshgoftaar T. M. (2019). Survey on deep learning with class imbalance. J. Big Data 6:27. 10.1186/s40537-019-0192-5

[B35] Kang D. Gweon H. M. Eun N. L. Youk J. H. Kim J.-A. Son E. J.. (2021). A convolutional deep learning model for improving mammographic breast-microcalcification diagnosis. Sci. Rep. 11:23925. 10.1038/s41598-021-03516-0 34907330 PMC8671560

[B36] Kohiruimaki M. Ohtsuka H. Hayashi T. Kimura K. Masui M. Ando T.. (2006). Evaluation by weight change rate of dairy herd condition. J. Vet. Med. Sci. 68, 935–940. 10.1292/jvms.68.935 17019062

[B37] Kuzuhara Y. Kawamura K. Yoshitoshi R. Tamaki T. Sugai S. Ikegami M.. (2015). A preliminarily study for predicting body weight and milk properties in lactating Holstein cows using a three-dimensional camera system. Comput. Electron. Agric. 111, 186–193. 10.1016/j.compag.2014.12.020

[B38] LeCun Y. Bengio Y. Hinton G. (2015). Deep learning. Nature 521, 436–444. 10.1038/nature14539 26017442

[B39] Li G. Liu X. Ma Y. Wang B. Zheng L. Wang M.. (2022). Body size measurement and live body weight estimation for pigs based on back surface point clouds. Biosyst. Eng. 218, 10–22. 10.1016/j.biosystemseng.2022.03.014

[B40] MacDonald J. M. McBride W. D. O'Donoghue E. Nehring R. F. Sandretto C. Mosheim R.. (2007). Profits, costs, and the changing structure of dairy farming. USDA-ERS Econ. Res. Rep. 47. 10.2139/ssrn.1084458

[B41] Na M. H. Cho W. H. Kim S. K. Na I. S. (2022). Automatic weight prediction system for Korean cattle using Bayesian ridge algorithm on RGB-D image. Electronics 11:1663. 10.3390/electronics11101663

[B42] Norton T. Berckmans D. (2017). Developing precision livestock farming tools for precision dairy farming. Anim. Front. 7:18–23. 10.2527/af.2017.0104

[B43] Nyalala I. Okinda C. Kunjie C. Korohou T. Nyalala L. Chao Q.. (2021). Weight and volume estimation of poultry and products based on computer vision systems: a review. Poult. Sci. 100:101072. 10.1016/j.psj.2021.101072 33752071 PMC8010860

[B44] Ou G. Murphey Y. L. (2007). Multi-class pattern classification using neural networks. Pattern Recognit. 40:4–18. 10.1016/j.patcog.2006.04.041

[B45] Ozkaya S. Bozkurt Y. (2008). The relationship of parameters of body measures and body weight by using digital image analysis in pre-slaughter cattle. Arch. Anim. Breed. 51, 120–128. 10.5194/aab-51-120-2008

[B46] Poncheki J. K. Canha M. L. S. Viechnieski S. L. Almeida R. D. (2015). Analysis of daily body weight of dairy cows in early lactation and associations withproductive and reproductive performance. Rev Bras Zootec. 44, 187–192. 10.1590/S1806-92902015000500004

[B47] Qi X. Dong J. Lan Y. Zhu H. (2022). Method for identifying litchi picking position based on YOLOv5 and PSPNet. Remote Sens. 14:2004. 10.3390/rs14092004

[B48] Qiao Y. Hu Y. Zheng Z. Qu Z. Wang C. Guo T.. (2022). A diameter measurement method of red jujubes trunk based on improved PSPNet. Agriculture 12:1140. 10.3390/agriculture12081140

[B49] Qiao Y. Kong H. Clark C. Lomax S. Su D. Eiffert S.. (2021). Intelligent perception-based cattle lameness detection and behaviour recognition: a review. Animals 11:3033. 10.3390/ani11113033 34827766 PMC8614286

[B50] Qiao Y. Truman M. Sukkarieh S. (2019). Cattle segmentation and contour extraction based on Mask R-CNN for precision livestock farming. Comput. Electron. Agric. 165:104958. 10.1016/j.compag.2019.104958

[B51] Quinlan J. R. (1986). Induction of decision trees. Mach. Learn. 1, 81–106. 10.1007/BF00116251

[B52] Robbins J. Von Keyserlingk M. Fraser D. Weary D. (2016). Invited review: farm size and animal welfare. J. Anim. Sci. 94, 5439–5455. 10.2527/jas.2016-0805 28046157

[B53] Ruchay A. Kober V. Dorofeev K. Kolpakov V. Dzhulamanov K. Kalschikov V.. (2022). Comparative analysis of machine learning algorithms for predicting live weight of Hereford cows. Comput. Electron. Agric. 195:106837. 10.1016/j.compag.2022.106837

[B54] Rumelhart D. E. Hinton G. E. Williams R. J. (1986). Learning representations by back-propagating errors. Nature 323, 533–536. 10.1038/323533a0

[B55] Russell B. C. Torralba A. Murphy K. P. Freeman W. T. (2008). LabelMe: a database and web-based tool for image annotation. Int. J. Computer Vision 77, 157–173. 10.1007/s11263-007-0090-8

[B56] Sant'Ana D. A. Pache M. C. B. Martins J. Soares W. P. de Melo S. L. N. Garcia V.. (2021). Weighing live sheep using computer vision techniques and regression machine learning. Mach. Learn. Appl. 5:100076. 10.1016/j.mlwa.2021.100076

[B57] Sheu M.-H. Morsalin S. S. Wang S.-H. Wei L.-K. Hsia S.-C. Chang C.-Y.. (2022). FHI-Unet: faster heterogeneous images semantic segmentation design and edge AI implementation for visible and thermal images processing. IEEE Access 10:18596–18607. 10.1109/ACCESS.2022.3151375

[B58] Stern U. He R. Yang C.-H. (2015). Analyzing animal behavior via classifying each video frame using convolutional neural networks. Sci. Rep. 5:14351. 10.1038/srep14351 26394695 PMC4585819

[B59] Storey G. Meng Q. Li B. (2022). Leaf disease segmentation and detection in apple orchards for precise smart spraying in sustainable agriculture. Sustainability 14:1458. 10.3390/su14031458

[B60] Tasdemir S. Urkmez A. Inal S. (2011). Determination of body measurements on the Holstein cows using digital image analysis and estimation of live weight with regression analysis. Comput. Electron. Agric. 76, 189–197. 10.1016/j.compag.2011.02.001

[B61] Wang D. He D. (2022). Fusion of Mask RCNN and attention mechanism for instance segmentation of apples under complex background. Comput. Electron. Agric. 196:106864. 10.1016/j.compag.2022.106864

[B62] Wang J. Sun K. Cheng T. Jiang B. Deng C. Zhao Y.. (2020a). Deep high-resolution representation learning for visual recognition. IEEE Trans. Pattern Anal. Mach. Intell. 43, 3349–3364. 10.1109/TPAMI.2020.2983686 32248092

[B63] Wang Q. Wu B. Zhu P. Li P. Zuo W. Hu Q.. (2020b). “ECA-Net: Efficient channel attention for deep convolutional neural networks,” in Proceedings of the IEEE/CVF Conference on Computer Vision and Pattern Recognition (Amsterdam: Elsevier Science Publishers B. V.). 10.1109/CVPR42600.2020.01155

[B64] Wathes C. M. Kristensen H. H. Aerts J.-M. Berckmans D. (2008). Is precision livestock farming an engineer's daydream or nightmare, an animal's friend or foe, and a farmer's panacea or pitfall? Comput. Electron. Agric. 64, 2–10. 10.1016/j.compag.2008.05.005

[B65] Weary D. M. Huzzey J. M. von Keyserlingk M. A. G. (2009). BOARD-INVITED REVIEW: USING behavior to predict and identify ill health in animals1. J. Anim. Sci. 87, 770–777. 10.2527/jas.2008-1297 18952731

[B66] Weber V. A. M. Weber F. L. Gomes R. C. Oliveira Junior A. S. Menezes G. V. Abreu U. G. P.. (2020a). Prediction of Girolando cattle weight by means of body measurements extracted from images. Rev. Bras. Zootec. 49. 10.37496/rbz4920190110

[B67] Weber V. A. M. Weber F. L. Oliveira A. S. Astolfi G. Menezes G. V. de Andrade Porto J. V.. (2020b). Cattle weight estimation using active contour models and regression trees Bagging. Comput. Electron. Agric. 179:105804. 10.1016/j.compag.2020.105804

[B68] Witte J.-H. Gerberding J. Melching C. Gómez J. M. (2021). “Evaluation of deep learning instance segmentation models for pig precision livestock farming,” in Business Information Systems (Hannover). 10.52825/bis.v1i.59

[B69] Woo S. Park J. Lee J.-Y. Kweon I. S. (2018). “Cbam: Convolutional block attention module,” in Proceedings of the European Conference on Computer Vision (ECCV) (Munich). 10.1007/978-3-030-01234-2_1

[B70] Wu F. Chen F. Jing X.-Y. Hu C.-H. Ge Q. Ji Y.. (2020). Dynamic attention network for semantic segmentation. Neurocomputing 384, 182–191. 10.1016/j.neucom.2019.12.042

[B71] Xu J. Lu K. Wang H. (2021). Attention fusion network for multi-spectral semantic segmentation. Pattern Recognit. Lett. 146, 179–184. 10.1016/j.patrec.2021.03.015

[B72] Yang Y. Ma J. Qu F. Ding T. Shan H. (2023). “Crop weed image recognition of UAV based on improved HRNet-OCRNet,” in Third International Conference on Artificial Intelligence and Computer Engineering (ICAICE 2022) (Wuhan). 10.1117/12.2671254

[B73] Yu H. Lee K. Morota G. (2021). Forecasting dynamic body weight of nonrestrained pigs from images using an RGB-D sensor camera. Transl. Anim. Sci. 5:txab006. 10.1093/tas/txab006 33659861 PMC7906448

[B74] Yuan X. Chen Z. Chen N. Gong J. (2021). Land cover classification based on the PSPNet and superpixel segmentation methods with high spatial resolution multispectral remote sensing imagery. J. Appl. Remote Sens. 15:034511. 10.1117/1.JRS.15.034511

[B75] Yuan Y. Chen X. Wang J. (2020). “Object-contextual representations for semantic segmentation,” in Computer Vision–ECCV 2020, 16th. European Conference, Glasgow, UK, August 23–28, 2020, Proceedings, Part VI 16 (Glasgow). 10.1007/978-3-030-58539-6_11

[B76] Zhang H. Wu C. Zhang Z. Zhu Y. Lin H. Zhang Z.. (2022). “Resnest: split-attention networks,” in Proceedings of the IEEE/CVF Conference on Computer Vision and Pattern Recognition (New Orleans, LA: IEEE). 10.1109/CVPRW56347.2022.00309

[B77] Zhang Y.-A. Sun Z. Zhang C. Yin S. Wang W. Song R. (2021). Body weight estimation of yak based on cloud edge computing. EURASIP J. Wirel. Commun. Netw. 2021, 1–20. 10.1186/s13638-020-01879-y

[B78] Zhao H. Shi J. Qi X. Wang X. Jia J. (2017). “Pyramid scene parsing network,” in Proceedings of the IEEE Conference on Computer Vision and Pattern Recognition (Honolulu, HI: IEEE). 10.1109/CVPR.2017.660

[B79] Zhao H. Zhang Y. Liu S. Shi J. Loy C. C. Lin D.. (2018). “Psanet: Point-wise spatial attention network for scene parsing,” in Proceedings of the European Conference on Computer Vision (ECCV) (Munich). 10.1007/978-3-030-01240-3_17

[B80] Zhao Y. Xiao Q. Li J. Tian K. Yang L. Shan P.. (2023). Review on image-based animals weight weighing. Comput. Electron. Agric. 215:108456. 10.1016/j.compag.2023.108456

